# Exploring Periostracum as an Alternative Root Canal Irrigant: Insights From Zebrafish Embryo Experiments

**DOI:** 10.7759/cureus.56638

**Published:** 2024-03-21

**Authors:** Annie Sylvea Valan, Jogikalmat Krithikadatta, Ajay Guru

**Affiliations:** 1 Department of Conservative Dentistry and Endodontics, Saveetha Dental College and Hospitals, Saveetha Institute of Medical and Technical Sciences, Saveetha University, Chennai, IND; 2 Department of Cariology, Saveetha Dental College and Hospitals, Saveetha Institute of Medical and Technical Sciences, Saveetha University, Chennai, IND

**Keywords:** biofilm eradication, antibacterial properties, root canal irrigants, novel periostracum irrigant, zebrafish embryos

## Abstract

Objectives

Root canal treatments aim to eliminate biofilms effectively. Considering the limitations of chemical irrigants, there is growing interest in natural alternatives like periostracum due to their antibacterial and fouling-resistant properties. This study aimed to assess periostracum’s toxicity as a root canal irrigant by investigating its effects on zebrafish embryos’ heart rate, survival rate, and hatching rate, as well as inflammation studies using neutral red assays comparing it to standard irrigants like ethylenediaminetetraacetic acid (EDTA), chlorhexidine (CHX), and sodium hypochlorite (NaOCl).

Materials and methods

Zebrafish embryos were exposed to varying concentrations of periostracum irrigant and standard irrigants. Heart rate, survival rate, and hatching rate were evaluated as indicators of developmental toxicity using microscopy. Statistical analysis, utilizing GraphPad Prism software (version 5.03, GraphPad Software, LLC, San Diego, California, United States), involved one-way ANOVA and Tukey’s post-hoc test to determine significance levels (p < 0.05) across control and other groups based on triplicate means and standard deviation.

Results

The periostracum irrigant demonstrated superior survival rates, heart rates, and hatching rates at specific concentrations compared to standard irrigants (p < 0.01), maintaining favorable heart rates and hatching rates at those concentrations. However, higher concentrations resulted in diminished hatching rates (p < 0.05). Additionally, this study revealed increased inflammation when larvae were treated with NaOCl, EDTA, and CHX. Conversely, no inflammation was observed when subjected to periostracum irrigants. These findings suggest potential advantages of periostracum as a root canal irrigant due to its increased biocompatibility.

Conclusion

Periostracum displayed promising attributes in zebrafish embryo experiments, such as stable heart rate, hatching rate, and survival rate, along with reduced developmental toxicity and inflammation, indicating potential advantages as a root canal irrigant, including reduced toxicity compared to conventional agents. Further research involving diverse demographics and long-term effects is crucial to validate periostracum’s clinical applicability and safety in endodontic therapies.

## Introduction

Zebrafish are frequently employed as model organisms in biomedical investigations owing to their genetic resemblances to humans, the transparent nature of their embryos, which enable convenient observation, and their rapid reproductive cycle [1]. Zebrafish have also been harnessed for toxicology inquiries owing to their heightened sensitivity to toxins and efficient absorption and dispersion of chemicals. Moreover, the zebrafish genome shares homologs with 70% of human genes, including over 80% of those linked to human diseases, enabling a more direct application of findings compared to studies involving invertebrates. This underscores the substantial investment in this species across various translational biomedical research domains [2]. While cell culture studies are commonly used for assessing cytotoxicity, zebrafish studies offer a more cost-effective alternative method to evaluate the embryotoxicity of substances. Utilizing zebrafish as a toxicity model involves exposing them to various chemicals or substances and then observing and recording alterations in their behavior, developmental trajectory, and survival outcomes [3]. Diverse metrics can be assessed, including alterations in heart rate, behavior, gene expression, and tissue integrity. These outcomes serve as indicators to ascertain the toxicity of the substance and potential hazards to human health. Zebrafish toxicity assessments have proven instrumental in appraising the toxicity of compounds and medications, alongside environmental pollutants like pesticides and heavy metals [4]. These evaluations can be conducted across multiple phases of the zebrafish life span, spanning from embryonic to adult stages, enabling investigations into both immediate and prolonged toxicity effects. On the whole, the zebrafish model serves as a valuable instrument for toxicity assessment owing to its heightened sensitivity, user-friendly nature, and capacity to unveil toxicity mechanisms. Nevertheless, akin to any model organism, there exist constraints in its application, demanding researchers to meticulously weigh the applicability of their discoveries in the context of human relevance [5]. The zebrafish model holds significant implications in various research domains, including dentistry. Within dental science, these fish serve as crucial subjects for investigating tooth development, regeneration, and ailments. Their unique ability to regenerate teeth throughout their lives offers a valuable window into comprehending the intricate mechanisms involved [6]. Investigating stem cell functions and unraveling genetic and signaling pathways are pivotal aspects of zebrafish-centered studies in tooth regrowth [7].

Zebrafish models contribute significantly to understanding dental issues such as tooth decay and periodontitis by mimicking these conditions, allowing researchers to assess their impact on tooth structure and function. These models serve as testbeds for potential remedies and therapies. In addition, zebrafish play a vital role in dental material and implant research, offering a platform to scrutinize the compatibility and potential toxicity of these materials. The zebrafish model holds immense promise for dental applications, shedding light on various aspects of tooth biology, regeneration, and diseases [8]. However, the translation of these findings into human relevance demands careful consideration, acknowledging the inherent limitations of any model organism.

Desirable characteristics of root canal irrigants encompass lubrication of instruments and canal walls, dissolution of organic and inorganic matter, antimicrobial properties, non-cytotoxicity, and preservation of dental microstructure [9]. Challenges related to these solutions include their limited reach into the apical third and intricate anatomical structures, reduced effectiveness in the presence of infected debris, constraints related to clinical application time, and potential toxicity to periapical tissues [10]. A biofilm is an intricately organized assembly of bacterial cells enveloped within a matrix, primarily composed of extracellular polysaccharides but also containing proteins and DNA [11]. These communities of microorganisms, tightly adhering to surfaces or each other, are resilient against antimicrobial agents due to the matrix’s barrier effect, slowing down drug absorption into bacterial cells compared to their individual, free-floating state. Biofilms also provide an environment conducive to genetic mutations, aiding the survival and resistance development of microbial cells [12]. In dentistry, combating biofilms is challenging, especially during root canal treatments aimed at eliminating residual tissues, microbes, and biofilm within the canal space. The goal of endodontic research is complete biofilm eradication [13]. Addressing the limitations of chemical antibacterial agents, there is a growing interest in herbal extracts for their biocompatibility and dental-friendly attributes. These natural products, with their anti-inflammatory, antimicrobial, and antioxidant properties, are being explored for canal disinfection in endodontics [14].

In consideration of the aforementioned aspects, an endodontic irrigant has been developed, aligning with the pursuit of combating biofilms and enhancing treatment efficacy. Our research employs zebrafish to assess toxicity, with a specific focus on evaluating the biocompatibility of periostracum as an irrigant. There is evidence in the existing literature highlighting the role of periostracum in the elimination of biofilms [15,16]. The upcoming phase involves evaluating the cytotoxicity of this novel irrigant, marking a crucial step in ensuring the safety and biocompatibility of this formulation for potential use in endodontic procedures. This study aimed to assess the toxicity of periostracum irrigant by examining its effects on zebrafish embryos’ heart rate, survival rate, hatching rate, and development, as well as inflammation, using the neutral red assay and comparing it to standard irrigants like ethylenediaminetetraacetic acid (EDTA), chlorhexidine (CHX), and sodium hypochlorite (NaOCl).

## Materials and methods

This study was conducted at Saveetha Dental College and Hospitals, Saveetha Institute of Medical and Technical Sciences, Saveetha University, Chennai, India. It has been performed with the approval of the Institutional Scientific Review Board (SRB/SDC/ENDO-2103/23/015).

Preparation of periostracum irrigant

The extraction process for periostracum commenced by gathering seashells from the coastal shores of Chennai, India. These shells were carefully subjected to a 1:2 ratio solution of vinegar and seawater within a 250-mL beaker, which was left undisturbed for 24 hours, following the method outlined by Grandison et al. [17] for optimal periostracum extraction without compromising the integrity of the shell. After the 24-hour duration, the extracted periostracum was delicately separated from the shell using sterile forceps and subsequently preserved in seawater. Any samples displaying shell damage or periostracum fouling were purposefully omitted from the study. To ensure cleanliness, the samples underwent a meticulous pre-submersion cleansing process to eliminate microfoulants, biofilms, and detritus. ASTM Type 1 quality HPLC-grade water was utilized to rinse periostracum peels, ensuring the removal of excess salts and acetic acid residues. Subsequently, the periostracum peels were freeze-dried at -80°C, followed by lyophilization (TSI Type Tray vacuum level 0.001 m bar, refrigerant CFC free), and then they were finely ground into a powder using an electric, food-grade grinder.

Solution Concentration Technique

Dissolving periostracum powder in distilled water involves precise measurements for each concentration level: 1 mg, 10 mg, and 20 mg in 1 ml of distilled water. The stirring method was employed to ensure homogeneity. This direct dissolution technique enables a controlled and accurate preparation process, ensuring the creation of distinct concentration levels crucial for the study’s investigative phases. This particular concentration of periostracum was chosen to investigate the potential correlation between increased periostracum concentration and heightened toxicity.

Origin and maintenance of zebrafish

Adult zebrafish (wild type; AB strain, aged four months) were procured from the NSK Aquarium in Kolathur, Tamil Nadu, India. Upon arrival, male and female fish were housed separately in our facility, maintaining a 10-L glass tank environment at 28.5˚C and a 14/10-hour light/dark cycle. They were fed thrice daily with live brine shrimp (*Artemia salina*). After a month-long acclimatization period, the fish were used for breeding purposes, and the embryos obtained were employed for subsequent experiments [18]. Collected embryos underwent microscopic analysis, discarding unfertilized ones and transferring fertilized embryos to a six-well plate for incubation in an E3 medium.

Developmental toxicity in embryos

Transparent zebrafish embryos are extensively employed in studying developmental toxicity, facilitating straightforward observation of defects and abnormalities during their rapid development. Within this investigation, the compounds employed to evaluate cytotoxicity encompassed saline (serving as the control) and various interventions: 17% EDTA, 2% CHX, and 3% NaOCl, alongside periostracum irrigant at concentrations of 1 mg, 10 mg, and 20 mg per ml. These chemical agents were administered to the zebrafish subjects for analysis. Zebrafish were typically exposed to the chemical agents by immersing them in solutions containing these substances for a specified duration of 24 hours [19]. Following treatment, the developmental stages of the embryos are observed under an inverted microscope to identify and examine potential malformations.

Survival rate in zebrafish embryos

Assessing the survival rate of zebrafish embryos is a prevalent method employed to evaluate the impact of various chemicals and environmental elements on their development and viability. These embryos represent a prominent model organism owing to their small size, transparent bodies, and rapid development. The technique involves subjecting zebrafish embryos to diverse concentrations of the tested substance, followed by continuous observation to determine the survival percentage at each concentration [20]. Surviving embryos were tallied daily, and any deceased embryos were promptly eliminated. The survival rate (%) of embryos and larvae was evaluated by observing key parameters, including egg coagulation, heartbeat, and the absence of spontaneous motility.

Heart rate in zebrafish embryos

Measuring the heart rate of developing zebrafish embryos constitutes a method utilized in heart rate analysis. Zebrafish embryos serve as a prevalent model organism for scrutinizing heart development and function, owing to their transparent bodies, enabling clear observation of heart development. Zebrafish embryos at 48 hours post-fertilization (hpf) are suitable for capturing the entire heartbeat frequency. In this procedure, the embryos were immobilized in a small agarose volume and positioned beneath a microscope equipped with a high-speed camera. This camera captured the heart movements, and dedicated software was employed to analyze the images and determine the heart rate [21].

Hatching rate in zebrafish embryos

The analysis of the hatching rate in zebrafish embryos involves assessing the proportion of embryos successfully hatching from their chorions and the protective outer shells surrounding developing embryos. Zebrafish embryos usually hatch between 48 and 72 hpf, and a reduction in hatching rate may suggest developmental abnormalities or exposure to toxins or stressors. In this procedure, embryo hatching rates were examined using a subset of embryos from each exposed group, including the control. Embryos were immersed in an embryonic medium for 24 hours that dissolved the chorion, facilitating hatching. Daily counts were conducted to document the number of hatched embryos, and any deceased embryos were promptly removed. In each treatment group, the number of hatched larvae was recorded, and the hatching rate (%) was calculated using the formula: hatched numbers/total exposed numbers × 100. The number of hatched embryos is then tallied and compared to the total used to calculate the hatching rate [22].

Neutral red assay in zebrafish embryos

In the domain of zebrafish larval research, the neutral red assay stands as a quintessential methodology employed for quantifying macrophage populations within live specimens, holding profound significance in discerning immune cell functionality and its pivotal role in safeguarding the organism against pathogens and exogenous entities. This assay, a well-established and widely embraced technique, serves as a cornerstone in the assessment of macrophage activity within zebrafish larvae. The experimental process entails immersing live larvae in a solution laden with neutral red dye, facilitating its uptake specifically by macrophages. Within these specialized immune cells, the dye accumulates within lysosomes, intricate cellular compartments responsible for the degradation and processing of foreign particles. Following incubation, a meticulous washing phase is employed to eliminate surplus dye, ensuing anesthesia of the larvae, and subsequent microscopic imaging using a light microscope (Olympus Corporation, Tokyo, Japan). Manifesting as distinctive red markers within the larvae, the macrophages are discerned and quantified utilizing sophisticated software, offering a precise enumeration of these crucial immune cells in each specimen [23].

Statistical analysis

The findings were displayed as the triplicate mean alongside the standard deviation. Statistical analysis was carried out using GraphPad Prism software (version 5.03, GraphPad Software, LLC, San Diego, California, United States). A one-way ANOVA was conducted to determine the significance levels between the groups.

## Results

Developmental toxicity in embryos

**Figure 1:**
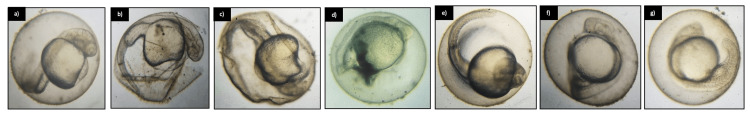
(a)-(g) demonstrate embryos treated with saline (negative control), 17% EDTA, 2% CHX, 3% NaOCl, 1 mg/ml periostracum irrigant, 10 mg/ml periostracum irrigant, and 20 mg/ml periostracum irrigant, respectively. Embryos were observed with normal morphology without any deformities when treated with different groups, as seen in (a), (e), (f), and (g). (b), (c), and (d) indicate developmental toxicity conditions in the embryos. In a research study, saline is used as a negative control. Saline, being a neutral and inert solution, is commonly used as a baseline or control substance to assess the specific impact of the experimental treatment. CHX, chlorhexidine; EDTA, ethylenediaminetetraacetic acid; NaOCl, sodium hypochlorite

In this study, zebrafish larvae were exposed to a range of irrigants for evaluation. The control group was treated with (a) saline, while the intervention groups encountered distinct substances: (b) 17% EDTA, (c) 2% CHX, (d) 3% NaOCl, and (e-g) periostracum irrigant at concentrations of 1 mg, 10 mg, and 20 mg per ml. The zebrafish larvae exhibited developmental toxicity when exposed to gold standard irrigants, such as NaOCl, CHX, and EDTA. This resulted in the deformation of the zebrafish embryo, leading to the manifestation of defective zebrafish larvae. The alterations observed in the larvae following exposure to these agents are visually depicted in Figure 1.

Heart rate in zebrafish embryos

**Figure 2:**
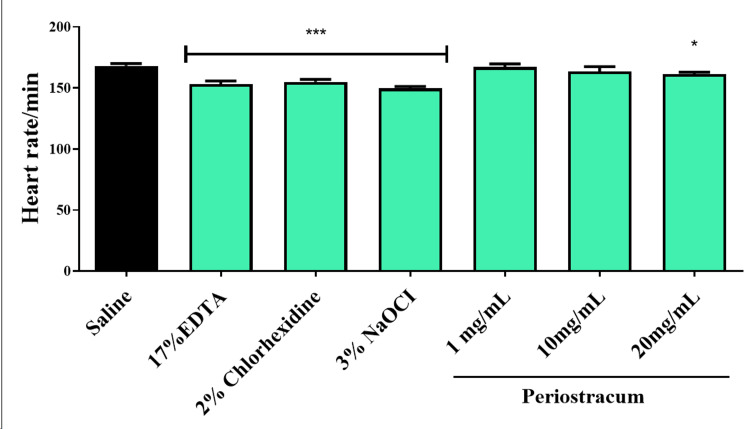
The heart rate of zebrafish embryos was investigated after being exposed to different groups for 24 hours. The *** (F value = 160.5; p < 0.01) and * (F value = 16.7; p < 0.05) indicate the significant difference between the control and intervention groups. EDTA, ethylenediaminetetraacetic acid; NaOCl, sodium hypochlorite

The embryonic heart rate was recorded for one minute under the microscope, and the average heart rate per minute was subsequently documented. The application of periostracum irrigant at concentrations of 1 mg/ml and 10 mg/ml did not yield a significant impact on the heart rates of zebrafish embryos compared to the control group. Conversely, a significant reduction in heart rate was observed when embryos were subjected to treatments involving EDTA, NaOCl, and CHX. Elevated concentrations of periostracum irrigant (20 mg/ml) resulted in observable changes in heart rates, as illustrated in Figure 2.

Hatching rate in zebrafish embryos

**Figure 3:**
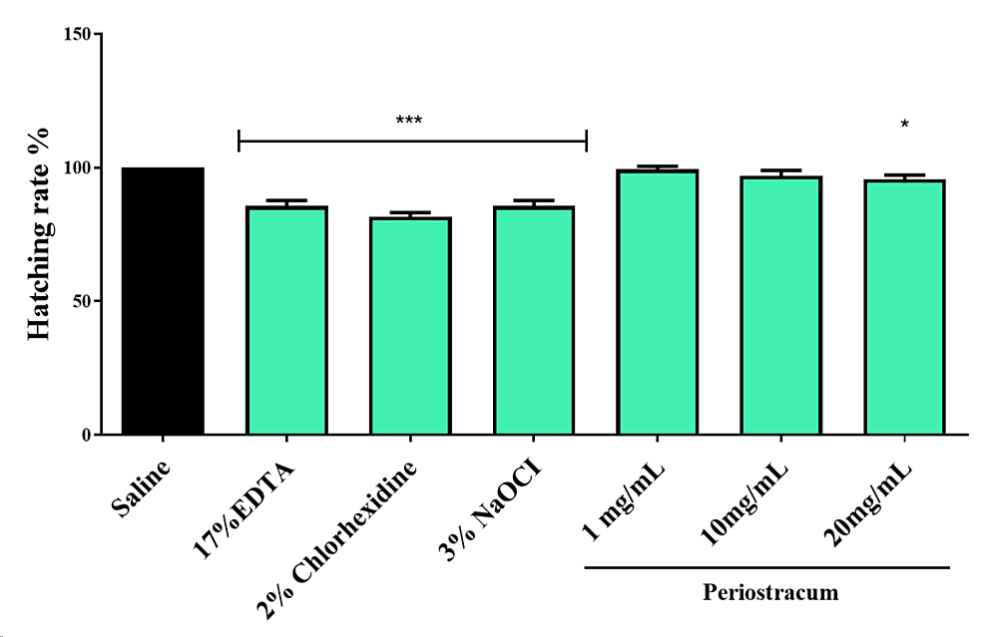
The hatching rate of zebrafish embryos was investigated after they were exposed to different groups for 24 hours. The *** (F value = 19.27; p < 0.01) and * (F value = 11.2; p < 0.05) indicate the significant difference between the control and intervention groups. EDTA, ethylenediaminetetraacetic acid; NaOCl, sodium hypochlorite

The analysis of hatching rates in zebrafish embryos stands as a significant avenue for understanding developmental nuances and the influence of environmental stressors. It acts as a crucial metric to assess the potential impact of various interventions. Notably, while EDTA, NaOCl, and CHX showcased a significant reduction in hatching rates (p < 0.01), periostracum irrigant demonstrated distinct changes only at higher concentrations, particularly at 20 mg/ml (p < 0.05), reflecting alterations in developmental outcomes compared to these chemical-based irrigants, as seen in Figure 3. Embryos treated with periostracum irrigant at concentrations of 1 mg/ml and 10 mg/ml demonstrated no significant impact on the hatching rates of zebrafish embryos when compared to the control group.

Survival rate in zebrafish embryos

**Figure 4:**
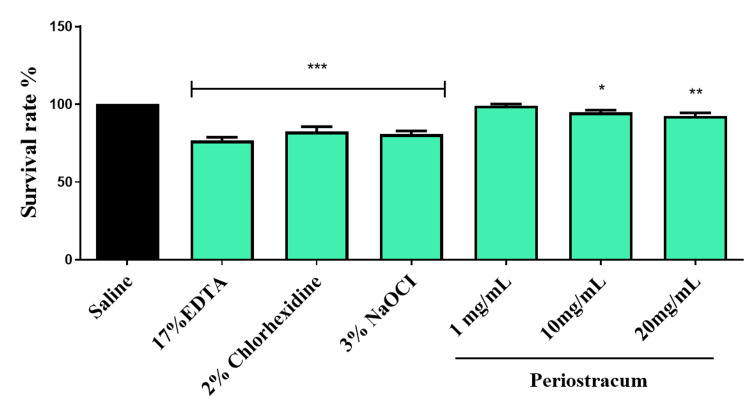
The survival rate of zebrafish embryos was investigated after being exposed to different groups for 24 hours. The *** (F value = 17.68; p < 0.01) and * (F value = 16.7; p < 0.05) indicate the significant difference between the control and intervention groups. EDTA, ethylenediaminetetraacetic acid; NaOCl, sodium hypochlorite

The survival rate analysis of zebrafish embryos revealed intriguing results regarding the impact of various substances on their viability. Notably, while EDTA, NaOCl, and CHX showcased decreased survival rates, 10 mg/ml and 20 mg/ml concentrations of periostracum irrigant also exhibited reduced survival rates (p < 0.05). However, even with decreased survival rates, the 10 mg/ml and 20 mg/ml periostracum irrigant groups displayed higher survival rates compared to EDTA, CHX, and NaOCl (p < 0.01). These findings suggest potential drawbacks associated with EDTA, CHX, and NaOCl in contrast to periostracum, highlighting periostracum’s comparatively higher survival rates, especially noticeable at the 1 mg/ml concentration (Figure 4).

Neutral red assay in zebrafish embryos

**Figure 5:**
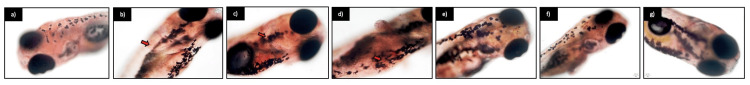
(a)-(g) demonstrate zebrafish larvae treated with saline (negative control), 17% EDTA, 2% CHX, 3% NaOCl, 1 mg/ml periostracum irrigant, 10 mg/ml periostracum irrigant, and 20 mg/ml periostracum irrigant, respectively. The macrophage migration in larvae due to toxicity conditions was observed through neutral red staining. The red arrow shows the macrophages in larvae, and the number of macrophages is presented in Figure 6. CHX, chlorhexidine; EDTA, ethylenediaminetetraacetic acid; NaOCl, sodium hypochlorite

**Figure 6:**
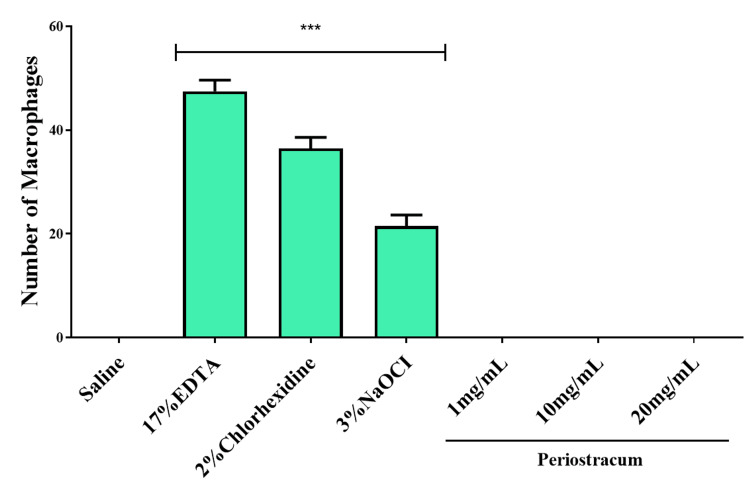
The number of macrophages evident in larvae due to toxicity was observed through neutral red staining and presented as a graph (F value = 21.58; p < 0.001). The number of macrophages evident in larvae due to toxicity was observed through neutral red staining and presented as a graph (F value = 21.58; p < 0.001).

In our study, zebrafish larvae were treated with different groups, such as (a) saline, (b) 17% EDTA, (c) 2% CHX, (d) 3% NaOCl, (e) 1 mg/mL periostracum irrigant, (f) 10 mg/mL periostracum irrigant, and (g) 20 mg/mL periostracum irrigant. Zebrafish larvae treated with NaOCl, EDTA, and CHX exhibited notably increased visibility of red stains, indicative of heightened macrophage activity compared to other groups, as observed in Figure 5. This elevation in staining suggests a potential activation or recruitment of macrophages in response to these chemical agents, hinting at an increased immune response or the presence of foreign substances that necessitate macrophage activity. Conversely, in the group treated with periostracum irrigant, we observed no stained macrophages. This could suggest a controlled and possibly more favorable immune response, hinting at a potentially less inflammatory reaction and a more harmonized interaction with the zebrafish larvae. These observations imply a differential impact of the substances tested on the zebrafish immune system. While NaOCl, EDTA, and CHX seemed to provoke a robust immune response, potentially indicative of heightened inflammation or reactivity, periostracum irrigant appeared to elicit a more tempered immune reaction, suggesting a potentially more favorable immune interaction profile (Figure 6).

## Discussion

Root canal irrigants play a crucial role in endodontic cleaning, falling into two categories based on their functions: those with antibacterial properties and those intended for decalcification. Commonly employed are NaOCl, citric acid, EDTA, and CHX. NaOCl stands as the predominant irrigant, typically used in concentrations of 0.5% to 5.25%. Its antimicrobial function, primarily attributed to HOCl (hypochlorous acid), makes it adept at dissolving necrotic pulp residues and dentinal collagen, yet it fails to eliminate the smear layer. Its in vivo efficacy diminishes due to biofilm and organic material presence, necessitating changes in concentration and continuous irrigation, although these practices could heighten side effects and olfactory challenges [24].

NaOCl, despite its efficacy, presents drawbacks, including its odor, toxicity, and inability to remove inorganic deposits from hard-to-reach anatomical regions like isthmuses and anastomoses, where mechanical cleansing is not feasible. CHX, effective at 2%, displays antibacterial prowess within the endodontic environment but cannot substitute NaOCl, lacking the capacity to eliminate necrotic residues or function optimally in the presence of organic remnants within the canal [25]. EDTA, while lacking antibacterial attributes, serves to eliminate the smear layer deposited by endodontic instruments, enhancing the accessibility of canal walls to disinfectants. However, inorganic residues that are not removed by NaOCl are eliminated through the use of an EDTA solution [26]. Challenges emerge from the extensive canal surface, leading to bacterial colonization within the dentinal tubules. The compacted smear layer in anastomoses, isthmuses, and tubule openings post-endodontic shaping comprises inorganic residues and biofilm, remaining resistant to typical root canal irrigants [27].

In earlier investigations conducted by Missotten et al., it was noted that all concentrations of NaOCl tested exhibited considerable cytotoxicity, resulting in the absence of viable ocular cells after a three-minute exposure to 0.5% NaOCl [28]. Sanchez et al. indicated that bactericidal concentrations of CHX demonstrated cytotoxic effects on canine embryonic fibroblasts [29]. Additionally, Li et al. illustrated that CHX has the potential to disturb the stable cellular redox balance, leading to the generation of free radicals and cell death. This implies cytotoxicity when extruded into periradicular tissues [30]. Findings from a study conducted by Karkehabadi et al. substantiated that the highest cytotoxicity was associated with EDTA, followed by QMix, CHX, and NaOCl. This pattern might be attributed to EDTA’s ability to reduce vasoactive intestinal polypeptide binding to macrophage membranes, which play a pivotal role in modulating the periapical immune response [31].

The utilization of periostracum irrigant as an alternative could stem from its potential advantages, such as biocompatibility with conventional root canal irrigants. Periostracum irrigants might present characteristics that address limitations observed in standard irrigants like NaOCl, EDTA, and CHX. The protective layer known as the periostracum, found on shells, plays a crucial role in deterring organisms that cause fouling. Fouling refers to the accumulation of unwanted materials on surfaces, often leading to a deterioration in performance or efficiency. Various anti-biofouling strategies exist, including the use of coatings with biocidal properties, creating smooth and less adhesive surfaces, or employing environmentally friendly alternatives to deter marine organisms. Research by Scardino et al. emphasizes the significance of an intact periostracum in reducing fouling activity [32]. Studies simulating microtopographical features akin to mussel shells have unveiled their capacity to discourage fouling, offering valuable insights into natural defense mechanisms [33]. Employing biomimicry to replicate such surfaces enables a deeper understanding of their impact, free from chemical or mechanical interventions. This underscores the imperative for comprehensive, prolonged investigations into the chemical anti-fouling properties of periostracum. These investigations should encompass a broader spectrum of commonly encountered oral microorganisms and extend to extensive field trials to thoroughly evaluate their potential applications.

The advantages of natural ingredients often lie in their reduced potential for adverse effects compared to chemicals. In the context of irrigants used in various applications, chemicals like NaOCl present a notable disadvantage due to the risk of hypochlorite accidents [34]. Conversely, natural ingredients generally lack such acute risks and offer a safer profile. This distinction emphasizes the significance of exploring alternatives like periostracum, which is derived from natural sources and might offer similar or enhanced effectiveness without the associated hazards common to chemical agents. Therefore, the pursuit of natural options not only aligns with the general principle favoring natural ingredients but also underscores the quest for safer, yet efficacious alternatives within the domain of oral health and related research [35]. In light of these considerations, exploring periostracum as a potential option gains prominence.

The limitations of this study encompass the restricted scope of evaluation, possibly confined to specific concentrations or application methods of periostracum. Additionally, the study lacks a comprehensive analysis of the long-term effects or broader implications of periostracum utilization in oral health interventions. Additional research endeavors are warranted to conduct comprehensive investigations into periostracum, specifically focusing on its impact in terms of antibacterial efficacy and antibiofilm efficacy. This will serve as the scope of future research.

## Conclusions

The study presents periostracum as a promising irrigant in terms of biocompatibility when compared to conventional chemical root canal irrigants. Utilizing zebrafish embryo experiments, periostracum demonstrated favorable attributes, notably enhancing the survival rates of embryos with minimal alterations in heart rate and hatching rates compared to standard irrigants such as EDTA, CHX, and NaOCl. These findings underscore the potential advantages of periostracum, including reduced toxicity, presenting a promising avenue for enhanced biocompatibility. However, the study’s scope is limited, necessitating further extensive research to validate its clinical applicability and establish its safety and efficacy in endodontic therapies.
